# Multi-Layer Reflectivity Calculation Based Meta-Modeling of the Phase Mapping Function for Highly Reproducible Surface Plasmon Resonance Biosensing

**DOI:** 10.3390/bios11030095

**Published:** 2021-03-23

**Authors:** Tzu-Heng Wu, Ching-Hsu Yang, Chia-Chen Chang, Hui-Wen Liu, Chia-Yu Yang, Tang-Long Shen, Chii-Wann Lin, Aurélien Bruyant

**Affiliations:** 1 Department of Biomedical Engineering, National Taiwan University, No. 1, Sec. 4, Roosevelt Rd., Taipei 10617, Taiwan; aresation@gmail.com (T.-H.W.); k8006780067@yahoo.com.tw (H.-W.L.); 2 Graduate Institute of Bio-Electronics and Bio-Informatics, National Taiwan University, No. 1, Sec. 4, Roosevelt Rd., Taipei 10617, Taiwan; d02945009@ntu.edu.tw; 3 Department of Medical Biotechnology and Laboratory Sciences, College of Medicine, Chang Gung University, Taoyuan 333, Taiwan; chang@mail.cgu.edu.tw; 4 Kidney Research Center, Department of Nephrology, Chang Gung Memorial Hospital, Taoyuan 333, Taiwan; 5 Department of Plant Pathology and Microbiology, National Taiwan University, No. 1, Sec. 4, Roosevelt Rd., Taipei 10617, Taiwan; r07633004@ntu.edu.tw (C.-Y.Y.); shentl@ntu.edu.tw (T.-L.S.); 6 Laboratory Light, Nanomaterials & Nanotechnologies (L2n), CNRS ERL 7004, University of Technology of Troyes, 12 rue Marie Curie, 10004 Troyes, France

**Keywords:** surface plasmon resonance biosensor, phase sensitive detection, exosome, algorithm

## Abstract

Phase-sensitive surface plasmon resonance biosensors are known for their high sensitivity. One of the technology bottle-necks of such sensors is that the phase sensorgram, when measured at fixed angle set-up, can lead to low reproducibility as the signal conveys multiple data. Leveraging the sensitivity, while securing satisfying reproducibility, is therefore is an underdiscussed key issue. One potential solution is to map the phase sensorgram into refractive index unit by the use of sensor calibration data, via a simple non-linear fit. However, basic fitting functions poorly portray the asymmetric phase curve. On the other hand, multi-layer reflectivity calculation based on the Fresnel coefficient can be employed for a precise mapping function. This numerical approach however lacks the explicit mathematical formulation to be used in an optimization process. To this end, we aim to provide a first methodology for the issue, where mapping functions are constructed from Bayesian optimized multi-layer model of the experimental data. The challenge of using multi-layer model as optimization trial function is addressed by meta-modeling via segmented polynomial approximation. A visualization approach is proposed for assessment of the goodness-of-the-fit on the optimized model. Using metastatic cancer exosome sensing, we demonstrate how the present work paves the way toward better plasmonic sensors.

## 1. Introduction

Surface plasmon resonance (SPR) has been widely used in various bio-sensing applications for its label-free detection capacity and high sensitivity. The advantages of SPR in biosensing make it an excellent candidate for medical applications such cancer diagnostic [1] and Tuberculosis diagnostics [2]. More recently, SPR has also taken up a vital role in the development of strategies against the SARS-CoV-2 global pandemic, such as the evaluation of SARS-CoV-2 mRNA vaccine efficacy [3] and evaluation of the inhibition effect of drugs on the spike protein [4]. In most cases, an SPR biosensor is realized in the form of a Kretschmann configuration [5], where a semi-infinite thin metal layer is sandwiched by a sensing medium on top and by a coupling prism on the bottom of the sensor chip. As light of specific wavelength, passing thorough the coupling prism at a fixed angle, becomes incidental to the metal thin film, the surface plasmon resonance occurs at the metal interface. At the resonance angle, the reflectance of the beam is reduced to minimum. The energy of the incident beam is converted into electromagnetic oscillation near the metal-medium interface, thereby making the resonance highly sensitive to the refractive index near the surface. As the refractive index changes, such as those induced by molecular binding events, the intensity and the phase of the incident p-polarized beam are altered correspondingly, making SPR a sensitive label-free sensor. The intensity and phase of the reflective beam can be described by complex Fresnel coefficient with multi-layer [6]. In each interface of the multi-layer, the complex reflectivity can be described as:

(1) $$r_{p}=\frac{n_{i}{\cos\theta }_{t}-n_{i+1}{\cos\theta }_{i}}{n_{i}{\cos\theta }_{t}+n_{i+1}{\cos\theta }_{i}}$$

where $r_{p}$ is the complex reflective coefficient at the interface, $n_{i}$ is the refractive index of the i^th^ material layer, and $\theta_{i}$ is the incident angle of the beam at the interface. $\theta_{t}$ is the refraction angle at interface between i and i + 1 material interface, which can be described by $\theta_{i}$ using Snell’s law with complex refractive index:

(2) $$n_{i}\sin\theta_{i}=n_{i+1}{\sin\theta }_{t}$$

The phase shift of the beam that is induced across i + 1^th^ layer can be described as:

(3) $$\Delta \phi_{i}=\frac{2{\pi h}_{i+1}{\cos\theta }_{t}n_{i+1}}{\lambda }$$

where $\Delta \phi_{i}$ is the phase shift across the interface, $\Delta \phi_{i}$ is the thickness of the $i+1^{\mathrm{th}}$ layer and λ is the wavelength of the incident beam. The reflection intensity and phase shift of the entire multilayer system can then be deducted via Equations (1)–(3), using Jones matrix.

Among the different SPR measurement methods, it is generally agreed that phase-sensitive SPR (pSPR) has better sensitivity over its intensity measurement counter-parts [7]. Typical resolution limit of pSPR has been reported between 10-7 RIU and 10-8 RIU [8,9]. The sharp non-linear transition near the resonance dip [10], together with low measurement noises (around ~0.001 rad in a careful setting), are two major rationales behind pSPR superior sensitivity.

Although the sensitivity of pSPR to subtle chemical changes on the surface is appreciable, this acute transducer response is also problematic. Since pSPR probes the polarization state of the beam under plasmon resonance conditions, the phase shift signal for a given biomolecular reaction can vary greatly depending on the thickness of the plasmon film as well as the working angle of the measurement. The more one asks to work at a film thickness optimized in terms of coupling efficiency, the more the phase shift of the pSPR as a function of the angle of incidence becomes non-linear and much sharper [11,12,13]. In other words, when considering the most commonly used fixed angle pSPR interrogation configuration, the measurement is highly subjected to film deposition variation as well as inaccuracy in the mechanical control of the angle of incidence. These reasons make it tedious and laborious to provide reproducible pSPR sensing signal [14], therefore it is difficult to enjoy sensitivity and reproducibility of pSPR at the same time. Unfortunately, sensorgram in form of phase, which is referred to as phasogram in present context, is still commonly used in pSPR measurements [13,15,16]. This may have been one of the key reasons behind the fact that intensity-based SPR is prevalent in industry despite the sensitivity advantage offered by pSPR. Based on the above discussion, the very key step toward a better pSPR biosensor is to secure sensitivity and reproducibility at the same time. There exist indeed one of the possible approaches to address such relevant issue. That is to experimentally calibrate the sensor phase shift as a function of effective refractive index detected by the sensor [17]. There are many advantages in building such mapping function and converting the measured phasogram to refractive index based sensorgram. Primary, all the calibrated sensorgram be free from the influence of film thickness variation and incidental angle variation. Secondly, as compared to the nonlinear pSPR phase response as a function of molecular weight, it is well known that refractive index increment is a linear function of molecular mass. Conventionally and most intuitively, a mapping function can be built from the experimental calibration data by the use of a simple non-linear approximation. However, within reasonable amount of calibration data point, the mapping function constructed by a simple regression cannot well portray the asymmetric curve of the pSPR. On the other hand, multi-layer reflectivity calculation based on the Fresnel coefficient is a well-established tool for study of the mapping function. If the most-fit multi-layer optical parameters can be allocated (i.e., precise film thickness, incident angle, optical constants, etc.), this tool can help to construct the mapping function from the very fundamental physical law. It would be nice therefore to use multi-layer reflectivity calculation as a trial function for optimization, and search for the optimized phase mapping function that corresponds to experimental data. Such use of multi-layer reflectivity calculation has nevertheless been limited by the fact that reflectivity calculation does not provide exact mathematical representation for a given model. Lacking exact form makes it impossible to use such model as an optimization trial function for finding of parameters of a pSPR sensor chip.

Here in this article, we intend to demonstrate a novel algorithm for building of pSPR phase mapping function. The proposed algorithm aims to address the difficulties in searching of phase mapping function. The use of the algorithm will allow pSPR to secure sensitivity and reproducibility at the same time, thereby paving way toward better pSPR applications. To the best of our knowledge, this is the first comprehensive methodology ever proposed to address such relevant issue. We will firstly describe the structure of the algorithm. The essence of the algorithm is to build phase mapping function out of experimental calibration data, on the basis of multi-layer Fresnel reflectivity calculation. In order to search for the optical parameters that best describe the empirical data, the algorithm adopts Bayesian optimization, using trial functions built from multi-layer model. The challenge here is to use multi-layer model, which has no discrete mathematical formulation, as trial function in optimization. This problem is addressed by meta-modeling of the multi-layer model during the optimization process. The meta-modeling, i.e., “modeling of a model”, of the multi-layer calculation is achieved by segmented polynomial approximation. Aside from addressing the critical issue in optimization, we also propose the use of visualization strategy in our algorithm, to evaluate the goodness-of-the-fit in this high dimensional optimization problem. To the best of our knowledge, our approach has not been mentioned elsewhere.

We will then stress the relevance of phase conversion in case of a fixed angle interrogation pSPR, through phasogram obtained from pSPR chips. To better demonstrate the efficacy of the algorithm, we take a step further to use organo-tropic exosome sensing as a study model. 4175 lung-tropic exosomes used herein are highly metastatic and invasive bio-nanoparticles, with size ranging from 50–150 nm. Our past studies have shown that such particles participate in long range cell–cell communications that facilitate cancer progression and organo-tropic metastasis [18,19]. The above-mentioned works pointed out that the organ specific metastasis could be guided by exosomal surface proteins integrin [18], using different surface integrins as road-map marker. Due to their clinical significance and the need for parallel imaging of several markers, pSPR sensing with improved reproducibility is of high relevance. We have therefore used such sensing data as a demonstration model, where integrin aptamers are applied as sensing probes.

## 2. Materials and Methods

### 2.1. pSPR Set-Up

**Figure 1:**
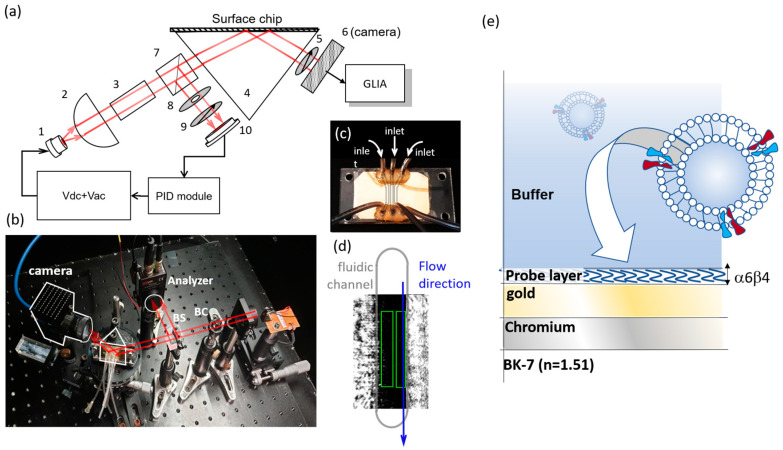
(**a**) the optical set-up of the pSPR system used in present work. (1) denotes the VCSEL source, (2) denotes the collimation lens which is a spherical lens. (3) is a YvO4 birefringent crystal, which creates a phase lag between p and s polarization, (4) is a coupling prism, (5) is a polarization analyzer, (6) is a camera, (7) is a beam splitter, (8) an adjustable iris, (9) is a polarization analyzer and (10) is a reference diode. (**b**) An image of the actual optical set-up. (**c**) SPR sensor chip coupled with the three independent micro Figure 1. and ROI 2 from left to right respectively. The gray line is a cartoon scheme of the microfluidic channel. Sample flows along the blue arrow direction, driven by a syringe pump. (**d**) The gray line is a cartoon scheme of the microfluidic channel. Sample flows along the blue arrow direction, driven by a syringe pump. (**e**) A cartoon scheme of sensor chip and corresponding five-layer model.

In order to optimize sensitivity, the pSPR chips used herein has a gold plasmonic layer with 48 nm nominal thickness. A 2-nm of chromium layer is used for better adhesion, and a BK-7 prism is applied for plasmonic coupling. The metal deposition was carried out using e-beam evaporator with 0.2 Å/s rate. The pSPR system was made of a phase modulation homodyne polarimetric interferometer optical scheme. A close view of the system is shown in Figure 1a,b. As shown by the Figure 1 is a vertical cavity surface emitting laser (VCSEL) source from Philips (ULM-852-BS-PL-S46FZP). (2) is a spherical lens for collimation of the incident source beam (LA1951 from ThorLab). The incident beam then passes through a birefringent crystal made of YvO4 crystal. The crystal created a phase lag between p and s polarization of the beam. After the incident beam reflected from the SPR chip through the coupling prism (4), the p and s polarization are projected into one polarization axis through the use of an analyzer (5). The overlapping of the p and s polarization creates polarimetric homodyne interference signal, which was then captured by the camera (6). Since VCSEL is highly sensitive to ambient temperature change, a beam splitter (7) was placed before the coupling prism to share part of the beam to a reference detector. Using a reference detector to monitor the phase of the beam, the system can then adjust the DC level of the VCSEL correspondingly to compensate the wavelength drift over time, using a PID function from LabViEW (National Instrument).

To extract the phase from such homodyne optical set-up, a phase modulation was driven by direct sinusoidal current modulation on VCSEL. The current modulation led to a non-negligible wavelength shift, which then led to the phase modulation. The current modulation, under residual power modulation of VCSEL [20], led to following form of interference time beating:

(4) $$I\left(t\right)\sim I_{0}\left(1+\mu \sin\omega t\right)\left[1+\mathrm{mcos}\left(\Delta \phi \left(i\right)-\phi_{\mathrm{SPR}}\right)\right]$$

where $I_{0}$ is the amplitude of the intensity modulation, µ is the fraction of AC to DC beam intensity of the laser (e.g., µ = I(t)_AC_/I(t)_DC_), $\phi_{\mathrm{SPR}}$ is the phase shift induced by SPR, Δϕ(i) is level of the phase modulation as a function of the driving current, *m* is the interference contrast, and ω is the modulation angular frequency. In present case, the phase difference is induced between p-polarization and s-polarization of the beam via the birefringent crystal. Then, the phase modulation is generated by modulation of VCSEL wavelength. Therefore, the Δϕ(i) in present set-up can be expressed as:

(5) $$\Delta \phi \left(i\right)=d\left[\frac{2\pi \left(n_{e}-n_{0}\right)L}{\lambda \left(i\right)}\right]$$

where $n_{e}$ is the refractive index of the extraordinary axis of the YvO4 crystal, $n_{o}$ is the refractive index of the ordinary axis of the YvO4 crystal, L is the length of the crystal and λ(i) is the wavelength of the VCSEL as a function of driving current. Length of the birefringent crystal is ~1 cm in present case. Under the case of sinusoidal current modulation, we can derive Equation (5) into:

(6) $$\Delta \phi \left(i\right)=-\frac{2\pi \left(n_{e}-n_{0}\right)\beta L}{\lambda_{0}^{2}}\mathrm{di}\left(t\right)$$

where β=dλ/di is commonly referred to as current modulation efficiency. The modulation efficiency, which is the intrinsic nature of the VCSEL, in present case is typically around 0.6 nm/mA. Considering di(t)=Δi sin(ωt), we can further modify Equation (6) into:

(7) $$\Delta \phi \left(t\right)=\left[-\frac{2\pi \left(n_{e}-n_{o}\right)\beta L\Delta i}{\lambda_{0}^{2}}\right]\sin\left(\omega t\right)=\Delta \phi_{a}\sin\left(\omega t\right)$$

where $\Delta \phi_{a}$ is the final phase modulation depth. 

As reported by our group previously [21], even under such sinusoidal phase modulation with residual power modulation, phase information can still be extracted under given phase modulation depth, using a generalized lock-in amplifier method (GLIA). Three major steps are necessary for phase extraction using GLIA from such set-up. First, the interference time beating must pass through a high pass filter to remove DC signal component. After DC filtering, interference signal in Equation (4) is modified into the following form:

(8) $$\begin{array}{c} \tilde{I\left(t\right)}\sim I_{0}[\mu \sin\omega t+\mathrm{mcos}\left(\Delta \phi_{a}\sin\omega t-\phi_{\mathrm{SPR}}\right)+m\mu \sin\omega t\;\cos\left(\Delta \phi_{a}\sin\omega t-\phi_{\mathrm{SPR}}\right)-\\ {\mathrm{mJ}}_{0}\left(\Delta \phi_{a}\right)\cos\left(\phi_{\mathrm{SPR}}\right)-{m\mu J}_{1}\left(\Delta \phi_{a}\right)\sin\left(\phi_{\mathrm{SPR}}\right)] \end{array}$$

Secondly, the $\Delta \phi_{a}$ should be tuned to 3.8317 rad, via the control of the current modulation. Finally, lock-in reference signals having the same harmonic composition with the interferometric signal must be used. Therefore, the reference signals take the following form:

(9) $$R_{X}=\cos\left(\Delta \phi_{a}\sin\omega t\right)=J_{0}\left(\Delta \phi_{a}\right)+2\sum_{n=1}^{\infty }J_{2n}\left(\Delta \phi_{a}\right)\cos\left(2n\omega t\right)$$

(10) $$R_{Y}=\sin\left(\Delta \phi_{a}\sin\omega t\right)=2\sum_{n=1}^{\infty }J_{2n-1}\left(\Delta \phi_{a}\right)\cos\left[\left(2n-1\right)\omega t\right]$$

where J_n_ denotes n-th Bessel function of first kind. With the pre-determined VCSEL modulation efficiency and the above-mentioned process, X and Y output of the GLIA have following form:

(11) $$\begin{array}{c} \;X=\frac{I_{0}m}{2}\{\cos\left(\phi_{\mathrm{SPR}}\right)\left[1+J_{0}\left(2\Delta \phi_{a}\right)-J_{0}^{2}\left(\Delta \phi_{a}\right)\right]+\\ \mu \sin\left(\phi_{\mathrm{SPR}}\right)J_{1}\left(2\Delta \phi_{a}\right)\} \end{array}$$

(12) $$\;Y=\frac{I_{0}m}{2}\{\sin\left(\phi_{\mathrm{SPR}}\right)\left[1-J_{0}\left(2\Delta \phi_{a}\right)\right]+\mu \cos\left(\phi_{\mathrm{SPR}}\right)J_{1}\left(2\Delta \phi_{a}\right)\}$$

After obtaining Equations (11) and (12), the phase can be then extracted by following the equation:

(13) $$\phi_{\mathrm{SPR}}=\arctan\left[\frac{\left[1+2J_{0}\left(2\Delta \phi_{a}\right)-J_{0}^{2}\left(\Delta \phi_{0}\right)\right]Y-{\mu J}_{1}\left(2\Delta \phi_{a}\right)X}{\left[1-2J_{0}\left(2\Delta \phi_{a}\right)\right]X-{\mu J}_{1}\left(2\Delta \phi_{a}\right)Y}\right]$$

where X and Y are the outputs of the GLIA lock-in amplifier. Limited by the scope and length of the present work, detailed derivation of GLIA signal processing of is not discussed herein. Interested readers can found information in our previous works [21,22,23]. The image of our sensor chip coupled with microfluidic channel can be seen in Figure 1c, with three independent microfluidic channels. An SPR intensity image is displayed in Figure 1d, where the gray line encircles the microfluidic channel area. The center of the image is of dark shade, since the image is taken at the SPR resonance dip. The channel area contacted by PMMA microfluidic and adhesive is revealed as bright zone in the image as it is far from the resonance dip. Two green squares indicate the typical ROIs that are selected for exosome detection as will be discussed later on. 

### 2.2. Algorithm 

**Figure 2:**
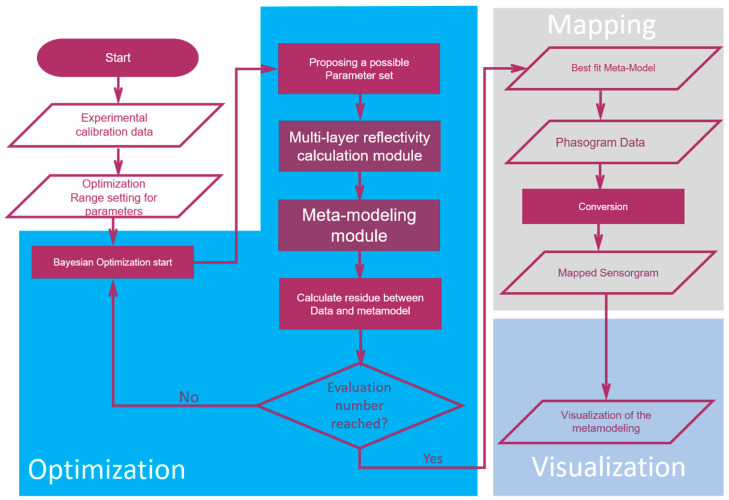
Algorithm flowchart.

The proposed algorithm is established with Python using Spyder IDE, following a linear process as shown in Figure 2. The algorithm is composed of three main processing stages: finding of optimized meta-model, data mapping and, finally, the production of visualization plot. A user-friendly interface is established using Tkinter package. As the initial input, the algorithm requires the experimental phase shift calibration data. These data were acquired by flowing reference solutions upon the sensor chip, with known refractive index. Reference solutions were made by adjusting the sodium chloride concentration in phosphate saline buffer in present context. Five-point calibration was used herein, which included four reference solutions and a baseline buffer data. After the input of sensor calibration data, the tuning range of optical parameters need to be designated before entering optimization module.

Based on experimental conditions herein, the calculation module is based on a five-layer reflection model (i.e., buffer, probe, gold, chromium, prism), with four input parameters for optimization. The cross-section scheme of the material stack used in this modeling process is shown in Figure 1e. The input parameters were incident angle, gold layer thick-ness, probe layer thickness and effective refractive index of the probe layer. The complete content of the code is provided as Supplementary Materials for interested reader, with a user instruction in the Supplementary Materials. Following experimental condition herein, reflectivity calculation is conducted under 850 nm wavelength. The gold refractive index was modeled as 0.16 + 5.34i. The refractive index of chromium adhesion layer was modeled as 3.24 + 3.49i. The coupling prism was made of BK-7 glass with 1.51 refractive index.

In each optimization iteration round, a trial optical parameter set is essayed by the algorithm to construct the phase mapping function using multi-layer reflective calculation module. Once the reflectivity calculation trial model is constructed, this data is sent for meta-modeling. In the meta-modeling process, the raw multi-layer model data is segmented into three sub-zones with similar data chunk size. After sectioning, these sub-zones are fitted with a third order polynomials individually. The meta-model is then built, in segmented form, using these polynomials. Note that the optimized phase mapping meta-models herein cover phase shift ranges slightly larger than that of the experimental data. In this way, extrapolation of the phase response is made possible. At the end of an iteration round, the sum of squared residuals of the meta-model is calculated and sent back to the optimization module. Based on residual information from each iteration round, the Bayesian optimization module can lead to a more efficient search over entire parameter spaces.

Once the designated iteration round is achieved, the phasogram is converted into final sensorgram in unit of effective refractive index change over time, using optimized meta-model. When the mapping finished, the algorithm provides sum of squared residuals visualization plot. We proposed herein such a visualization plot, where color heat map, data point size, x axis, and y axis are used to present our four fitting optical parameters in a given iteration, while z axis height is used to indicate the sum of squared residuals level as will be shown later. This visualization plot comprehensively depicts sum of squared residuals for all the trials, packed into a 3D space, despite that we are optimizing in four-dimension parameter space. This makes the assessment of goodness-of-the-fit much easier.

### 2.3. Preparation of Exosome Sample 

The 4175-LuT exosomes are obtained from human breast cancer cell line as previously reported by our colleagues [18]. The cell line was cultured in Dulbecco’s Modified Eagle’s medium (HyClone) with 10% FBS (Gibco, Thermo Fisher Scientific), L-glutamine 2 mM (HyClone), and antibiotics solution (HyClone) of 100 U/mL penicillin G, 100 g/mL streptomycin, and 0.25 g/mL amphotericin B. Cells were maintained in a humidified incubator with 5% CO_2_ at 37 °C. The petri-dishes were routinely tested for mycoplasma by EZ-PCRTM mycoplasma detection kit (Biological Industries) and were found to be negative. Around 10^6^ cells were seeded with 15 mL culture medium (with exosome-free FBS) in 150 mm culture plates for 3–4 days. When cell density reaches around 80%–90%, the exosomes are ready to be harvested. The isolation method was based on ultra-centrifugation method as reported by our colleague previously [24]. The ultra-centrifugation sorts out extracellular vesicle with larger particle sizes such apoptotic body or large micro-vesicles. First, the conditioned media were collected and spun by 500× *g* for 10 min at 4 °C. The supernatant was then collected and spun again at 3000× *g* for 10 min at 4 °C. We then extracted again the supernatant and re-spun at 12,000× *g* at 4 °C for 10 min. The final supernatant was then filtrated by a 0.22 μm filter. Subsequently, the filtered media were transferred to centrifuge tube and spun by 30,000 rpm with a centrifuge rotor (Beckman type 45Ti) for 16 h. The resulting pellet was then re-suspended in PBS. The obtained re-suspension was spun again by 30,000 rpm for another 8 h. The supernatant was discarded, and exosomes were then collected by re-suspending the pellet with 100–200 μL PBS.

### 2.4. Sensor Surface Modification and Biosensing Protocol 

To detect 4175-LuT exosome, an aptamer probe IDAB is used herein as reported by Berg et al. [25]. This aptamer probe targets the exosomal surface integrin sub-unit α6β4. The probe was synthesized by Purigo, Taiwan, with 5′ end thiolated for chip surface modification. The sequence of the IDAB probe is 5′-CGT-GCG-TAT-TCG-TAC-TGG-AAC-TGA-TAT-CGA-TGTCCC-3′. A 10 thymine block is added to 5′ end of the aptamer, therefore giving a final aptamer sequence of HS-5′-TTTTTTTTTT-CGT-GCG-TAT-TCGTAC-TGG-AAC-TGA-TAT-CGA-TGT-CCC-3′. A cartoon scheme for the modified gold chip is shown in Figure 1e, with IDAB secondary structure simulated using NUpack web server (http://www.nupack.org/partition/new, accessed on 23 March 2021) under experimental ionic strength condition. Based on the secondary structure, we predict that the probe layer has a thickness around 22 base pairs. This physical constraint was imposed on Fresnel modeling and optimization process herein. To provide proper spacing between the probe thereby maximizing the reaction rate, a spacer nucleic sequence is used (HS-5′-TTTTTTTTTT-3′). The mixing ratio between the aptamer and spacer sequence is 5:95 (v/v%) as optimized through empirical experiment. The surface modification process was carried out under 1x phosphate saline buffer (1x PBS) with 1M NaCl to reduce repulsion between highly negative nucleic acid phosphate backbone at vicinity, facilitating higher modification density. The surface modifications were undertaken for an hour under the pSPR monitoring, with a modification density similar to other report [26]. The exosome sensing is carried out under 1x PBSt condition (0.005% Tween-20 added in 1x PBS) to reduce non-specific binding [27]. In present work, Anti-CD9 is used as a secondary antibody to verify the binding authenticity of exosome by IDAB on SPR sensor chip. CD9, among other tetraspanin protein, is a transmembrane protein abundantly presented on 4175-LuT exosome. Therefore, the binding of the Anti-CD9 is commonly used to confirm the presence of the exosome.

### 2.5. Characterization of Exosome and the Aptamer Probe

**Figure 3:**
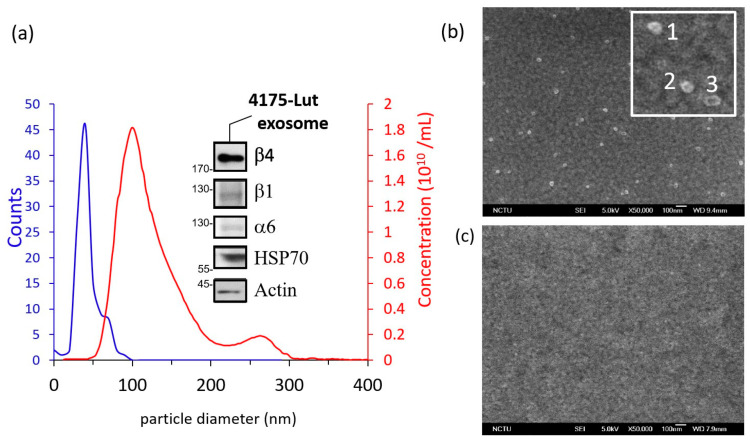
(**a**) The statistical data on exosome particle size both from SEM and NTA. Inset: the result of the Western blotting on the isolated exosomes. (**b**) SEM image of the exosome captured on IDAB modified gold surface. The inset is a magnified image of the exosome under SEM, showing 3 exosomes. (**c**) SEM image of a controlled surface, where exosome are not observed on the substrate.

The isolated exosome sample were carefully characterized by their surface protein content as well as via their physical properties. The surface markers were firstly examined with Western blotting method. The isolated exosomes with 20–30 g of total protein were prepared in sodium dodecyl sulfate (SDS) sample buffer. The sample was heat to 95 °C for 10 min before loaded on gels. The sample was then separated on 8–10% SDS-PAGE gel. Nitrocellulose membranes (NC membrane) were then used for transferring. After transferring, the NC membranes were blocked for 1 h in 5% non-fat milk in TBS buffer. Primary antibodies in Tris-Buffer Saline (TBS) with 1% Bovine Serum Albumin (BSA) was used for staining for overnight. For chemiluminescence detection of proteins, HRP-conjugated anti-rabbit IgG (Jackson Laboratory) and anti-mouse IgG (Jackson Laboratory) secondary antibodies, and ECL West-ern Blotting Detection Reagents (GE Healthcare) were used. The marker proteins for 4175-LuT exosomes were identified in the blotting (*Cf.* inset of Figure 3a), the protein panel was consisted of α_6_, β_1_, β_6_, HSP70 and Actin. As expected, integrin α6 and β4, which features the metastatic capacity of the 4175-LuT exosome, were identified in the blotting among loading control actin/HSP-70. The result verified that the extracted exosomes were indeed from the 4175-LuT cell line. The diameter of the isolated exosome, which is highly related to the biological origin of these particles, were characterized by Nano-particle Tracking Analysis system (NTA). The NTA system used herein was Nanosight NS300 from Malvern of United Kingdom. Based on the NTA measured data, the diameter of extracted bio-particles was centered around 120 nm (*Cf.* the red trace in the Figure 3a). This corresponds to previous reported size for exosomes [28]. Combining NTA data with Western blotting, we confirmed that the 4175-LuT exosomes were successfully extracted. Using the NTA system, the concentration of the extracted exosomes is estimated. Typically, extracted exosome concentration was between 10^10^–10^11^/mL. The extracted exosome is then diluted by 1x PBSt to the working concentration, which is set at 1.5 × 10^6^/mL. This concentration was selected since it is meaningfully challenging, considering the fact that the exosome concentration in the human body is around 10^8^–10^9^/mL [29]. Meanwhile, the dilution also reduces the background proteins from conditioned media.

To confirm the binding capacity of reported IDAB aptamer probe with the isolated 4175-LuT exosome, SEM investigations were carried out. For SEM imaging of exosomes, the sample preparation and processing methods were adapted from WU et al. [30]. In this experiment, IDAB modified pSPR chips were used as substrates. Controlled group pSPR chips were prepared with the surface passivated by thiolated poly-thymine. For both IDAB modified chip and control group, 10^8^/mL exosome sample was dispensed onto the substrate for capture in 0.005% PBSt. After a 1-hour reaction, the samples were rinsed again with running buffer to remove loosely bound exosomes. Before SEM imaging, the substrate was dipped in 2% paraformaldehyde to fix the exosome membrane. The substrate was then rinsed thoroughly with DI water, blown dry with nitrogen and then coated with ~10 nm of platinum as conductive layer. Imaging is carried out using Hitachi S-4700/a JEOL JSM-7600F SEM, under low beam energies (5.0–10.0 kV). The SEM image reveals that many exosomes were captured on the IDAB anchored surface. With the magnified image, we can see that exosome has the characteristic center depression under vacuum. Further particle tracing using Feret diameter method by ImageJ reveals that the particles have rather similar size distribution to NTA data, while minor size reduction can be observed (*Cf.* the blue trace in the Figure 3b). Both size reduction and center depression were expected due to vacuum imaging condition of SEM. In contrast to the IDAB modified surface, the reference surface is nearly free of exosomes. These results confirm the IDAB binding capacity and specificity towards 4175-LuT exosome.

## 3. Results

### 3.1. Revisit pSPR Sensing Phasogram

**Figure 4:**
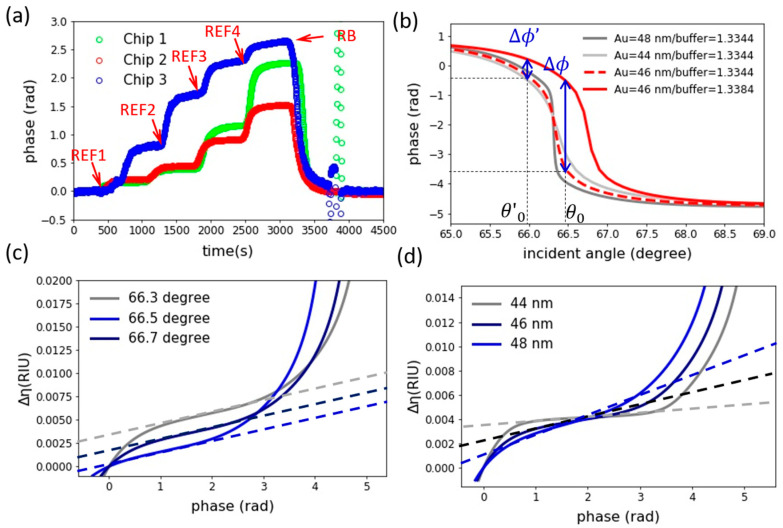
(**a**) sensorgram of pSPR chips upon different reference solutions. (**b**) Angle resolved phase spectrum of SPR simulated under five-layer Fresnel modeling. (**c**) The mapping function established via the five-layer Fresnel modeling under a fixed gold film thickness of 46 nm, with varying incident angle from 66.3 to 66.5 degree. (**d**) A mapping function established under fixed incident angle, with film thickness varies from 44 nm to 48 nm.

In this section, we demonstrate how pSPR phasogram may lead to seemingly different sensing results. Figure 4a demonstrates measurements from three individual IDAB modified sensor chips, upon a series of reference solutions. In this experiment, reference solutions with refractive index ranging from 1.3342, 1.3346, 1.3349, to 1.3364 were prepared by 1x PBS with 10 mM NaCl (REF1), 25 mM NaCl (REF2), 50 mM NaCl (REF3), and 100 mM NaCl (REF4) respectively. Judging from the data, the sensor chips developed different phase shifts towards the reference standards. While chip-1 and chip-3 developed relatively large phase shift, chip-3 is less sensitive and provides more of a nominal linear response.

The reason behind the seemingly different sensing response can be easily comprehended from Figure 4b. In this figure, we computed a series angle resolved SPR phase response by five-layer Fresnel reflective model, as detailed in the methodology section. The red dashed line indicated the pSPR angle response for a sensor chip with 46 nm film thickness. As shown by the figure, Δϕ can be measured at an incident angle of $\theta_{0}$, if refractive index of sensing median is to change from 1.3344 to 1.3384 as shown by the multi-layer reflection simulation result. However, if the source beam incident angle is to off-set down to $\theta_{0}^{\prime }$, the measurement phase shift dramatically reduced to $\Delta \phi^{\prime }$. This indicates how incident angle can greatly alters the phase shift data, even if identical sensor chip and sample are used in a given measurement. Meanwhile, the difference in plasmonic film thickness also alters how phase shifts upon a given refractive index change. As indicated by the grey line (44 nm gold thickness) and dark grey line (48 nm gold thickness), the slope of pSPR angle spectrum is strongly altered by variation in film thickness. Therefore, the alteration of film thickness changes how chip responds to external refractive index.

The two above factors combined lead to seemingly large variations in measurement. Moreover, fine control of these parameters across measurements is a non-trivial work. For example, it would be tedious to maintaining sub-nanometer film homogeneity across different fabrication batches or even across large sensing area. This why we propose the algorithm, which is designed to make pSPR sensing data more reliable without overly tedious work.

Using multi-layer Fresnel modeling, the phase-to-refractive index mapping function under various experimental conditions are shown in Figure 4c,d. These sensor response curves clearly reveal the non-linearity of pSPR toward surface refractive index change, and well demonstrated why raw pSPR data is too sophisticated for direct sensing data interpretation.

Under a fixed film thickness, we can see that incident angle alters the positioning of the sensor response along the mapping function. On the other hand, when film thickness increases with incident angle fixed, slopes of the mapping function is altered. As plasmonic film approaches optimized film condition, the sharpest sensor response can be found. When film thickness and angle vary together, this results in a variety of mapping functions, thereby giving us the seemingly different phasogram results across measurements.

### 3.2. Meta-Model and Mapping Results

**Figure 5:**
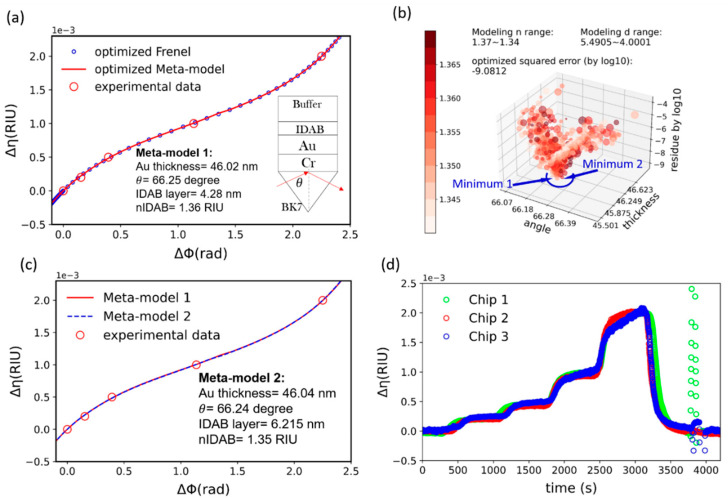
(**a**) Meta-model mapping functions corresponding to chip-1 in Figure 4a. In the figure, the red curve represents the optimized meta-model, the red circles represent the experimentally measured data and the blue circles represent the corresponding Fresnel model. (**b**) Proposed visualization chart for the meta-model 1. (**c**) Comparison of meta-model 1 and meta-model 2. (**d**) The sensorgram of the chip-1 to chip-3 after the conversion by the algorithm.

Figure 5a reveals the algorithm derived meta-model for the chip-1 shown in Figure 4a. As shown in the Figure 5a, the optimized multi-layer model (blue circles) fits nicely with experimental data (red circle). It is also clear from figure that the meta-model (red trace) is a good representation of the multi-layer model. With the aid of the proposed algorithm, meta-model is extended beyond the sensor calibration range. The optical parameters for the meta-model are shown in the inset of the Figure 5a.

Figure 5b is the visualization chart for the optimization process of the chip-1. In the visualization chart that we proposed herein, each scatter data spot represent a specific iteration round of the optimization process. The z-axis value indicates the sum of squared error in common logarithm scale, which is used as one of the indicators for goodness-of-the-fit. We define the value of the four fitting parameters in a given iteration by x value (incident angle), y value (thickness of the gold), color shade (refractive index of the probe layer) and the scatter spot size as the probe layer thickness.

As indicated by the figure, the meta-model 1 in Figure 5a reaches a sum of squared residuals logarithm of −9.0812, indicating high goodness-of-the-fit. In terms of evaluating goodness-of-the-fit, the visualization chart offers more than just squared error value. In this specific case, we can observe the funnel shape contour formed by sum of squared residuals logarithm data in the chart. This funnel shape indicate error minimum can be allocated. At the same time, it is clear from the image that more than one local minimum with similar error level can be found. The visualization data, asides from error value, provides insights into the existence of other possible model for the experiments under study.

At this point, we must explore how the propagation-of-error varies among the equivalent meta-models. Using the algorithm, an alternative meta-model (noted as model 2) with thicker IDAB layer and lower refractive index was generated. This is done by adjusting parameter tuning range of the optimization. The resulting model-1 and model-2 are compared and shown in Figure 5c. The figure reveals that these equivalent models, despite differences in parameters, present nearly identical formulation within the proposed working range. We therefore conclude that the equivalent models provide good mapping results, despite variations in parameter traits. The presence of the equivalent model is due to the fact that the nature of the modified molecular layer cannot be uniquely decided under a single wavelength SPR system as shown elsewhere [31]. In this perspective, the visualization chart can help users to note the importance of setting physical constraints in the optimization process or to be aware of the limitation of the meta-model.

Using established meta-model, all phasogram in Figure 4a are converted into refractive index based sensogram and shown in Figure 5d. The result indicates that three sensorgram are now highly consistent, once they are converted in refractive index via our algorithm.

### 3.3. Application to Lung-Tropic Metastatic Exosome Detection

In this section, we use 4175-LuT exosome detection to demonstrate the proposed algorithm. The main purpose of the demonstration is to show the efficacy of the method via image-based phase detection under a realistic sensing process, showing how the proposed method can provide highly consistent measurements across different sensing areas of a given sensor chip.

**Figure 6:**
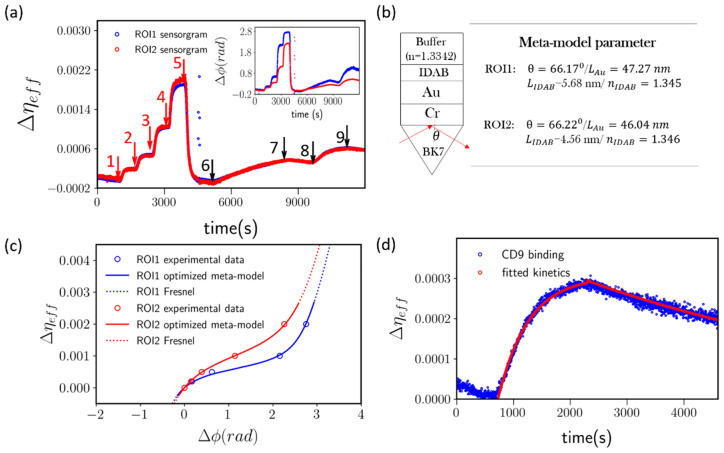
(**a**) 4175-LuT exosomal surface integrin α_6_β_4_ detection sensorgram, obtained using IDAB modified SPR chip. The blue trace is the sensorgram collected from ROI1 of microfluidic channel and red trace is collected from ROI2 for comparison. Inset: the phasogram of the exosome detection process before processed by the proposed algorithm. (**b**) The optimized meta-model parameters obtained for the ROIs under study. (**c**) The meta-models obtained for the ROIs under Scheme 1. red circle represent the data from ROI2. The meta-model is noted in blue line for ROI1, and red line for ROI2. The optimized Fresnel model is plotted in blue dotted line (ROI1) and red dotted line (ROI2). (**d**) CD9 sensorgram from ROI1 in blue trace and fitted Langmuir model in red trace.

The result of the measurement is shown in Figure 6a. In this set of experiment, two independent Region-Of-Interest (ROIs) within a single microfluidic channel are selected to monitor the detection process (*Cf.* Figure 1d from Methodology section). The chip surface was modified with IDAB aptamer probes for capturing the exosome via surface integrin α_6_β_4_. Converted sensorgram is shown in Figure 6a, and the inset reveals the phasogram before conversion. As indicated by the figure, the sensor was firstly calibrated with reference solutions as indicated by red arrow 1 to 4 with increasing refractive index respectively. After the calibration process, the sensor is flushed again with running buffer to establish baseline (t ~ 4500 s) (arrow 5). The 4175-LuT exosome was then introduced into the channel at a concentration of 1.5 × 10^6^/mL as indicated by the black arrow 6. The SPR signal increased correspondingly as exosomes arrived and bounded with the surface anchored IDAB probes. After 3000 s of the reaction time, the sensor is flushed with running buffer to confirm the strength of the exosome binding (black arrow 7). As shown by the figure, the binding between IDAB and exosomal surface integrin is strong as indicated by the slow decaying kinetics. By comparing the phasogram in the inset with the converted sensorgram, it is evident that raw phasogram lead to seemingly different sensor response despite such two ROIs are in close vicinity within the microfluidic channel (noted as ROI1 and ROI2 respectively). The fitting parameters for the mapping function are shown in Figure 6b and the mapping function can be found in Figure 6c. Based on these effective meta-models, we can see that the phasogram exhibit clear differences due to the fabrication and measurement conditions, even if the film thickness differs by only 1.2 nm and incident angle differs by only around 0.05 degree. Therefore, the mapping function is strongly needed to fully quantify pSPR sensing data.

To further validate the exosome binding event here, we introduce anti-CD9 antigen as secondary antibody at a concentration of 1 µg/mL. Anti-CD9 is introduced onto the surface as indicated by arrow 8 in Figure 6a and the sensor surface was subsequently washed with running buffer as indicated by arrow 9. The binding of anti-CD9 demonstrates a characteristic Langmuir binding curve. For the binding process, the signal can be described as:

(14) $$\frac{\Delta S\left(t\right)}{{\Delta S}_{\mathrm{Max}}}=1-e^{-\tau t}$$

where ΔS(t) is the sensor signal response as a function of time, τ is the half-life of the reaction and ${\Delta S}_{\mathrm{Max}}$ is the equilibrium signal level of the given reaction. The τ of the reaction is a function of Anti-CD9 protein concentration (C), having the form k_on_C + k_off_, where k_on_ is the kinetic constant of the forward reaction and k_off_ is the kinetic constant of the reverse reaction. On the other hand, the dissociation kinetics can be fitted with:

(15) $$\Delta S\left(t\right)={\Delta S}_{0}e^{-k_{\mathrm{off}}\left(t-t_{0}\right)}$$

where $t_{0}$ denotes the starting time of the dissociation and $\Delta S_{0}$ is the starting signal level of the dissociation. The fitted kinetic curve is indicated as red line in Figure 6d. Based on Equations (14) and (15), the τ of the anti-CD9 binding is 0.00188 s^−1^, k_on_ = 4.28 × 10^4^ M^−1^s^−1^ and koff = 0.000175 s^−1^. Combining the value of k_on_ and k_off_, the K_D_ of the binding is 3.65 nM for the anti-CD9 and CD9 interaction measured herein.

## 4. Conclusions

In this work, we demonstrate why a pSPR phasogram must be mapped into the refractive index based sensorgram in order to simultaneously enjoy the benefit of pSPR superior sensitivity and high data reproducibility. Conventionally, the mapping of the phase shift into refractive index space is a non-trivial and tedious work. In search of such mapping function, the most intuitive method is a simple non-linear fit to the experimental calibration data. Nevertheless, such a non-linear fit cannot nicely portray the asymmetric phase response curve. On the other hand, the multi-layer Fresnel calculation is a powerful tool in addressing such need, as it can build precise mapping function from very fundamental law. However, such multi-layer Fresnel calculation is not suited for optimization, since the calculation result has no exact mathematical formulation. To the best of our knowledge, there is no well-established methodology toward this relevant issue.

To this end, we have therefore proposed an algorithm to address such issue. The core of the algorithm is composed of optimization module, mapping module and visualization module. In order to apply Fresnel multi-layer model as our trial function in optimization, the trial Fresnel model is made into meta-model via segmented polynomial approximation. Each trial function is a Fresnel model made up of three segments. These segments are of similar data chunk size and approximated by a third order polynomial. The meta-models are then presented by these segmented polynomials.

Five-layer modeling is used herein with four parameters for optimization. The parameters are incident angle of measurement, gold film thickness, probe layer thickness and effective refractive index of the probe layer. In present case, the parameter optimization is a high dimensional optimization problem. A visualization strategy is applied to demonstrate the goodness-of-the-fit in our algorithm. The visualization chart provides not just sum of squared residuals of a given trial. The contour of the visualization plot also helps user to be aware of other possible equivalent meta-models. This provides a comprehensive perspective in goodness-of-the-fit, when we try to optimize in multi-dimensional parameter space. Although four-parameter optimization is demonstrated herein, it is possible to modify the algorithm to adapt to fitting problems with high dimension or different set of parameters.

Using a polarimetric phase modulation pSPR system, three different sensor chips are used to demonstrate how minor optical parameters differences can lead to seemingly different phasogram, even in the case of reference solution sensing with a known refractive index. Through the multi-layer modeling based on Fresnel coefficient, we show how film thickness and incident angle variation can have impact on resulting phasogram. We then reveal that, using the meta-modeling algorithm, highly consistent pSPR sensing results can be obtained. Using 4175-LuT exosome as a study model, we demonstrated the use of the algorithm in a relevant sensing application. The existence of the exosome in our sample is verified by nano-particle tracker, Western blot and scanning electron microscopy. IDAB aptamer probes was modified onto the pSPR sensor chip for detection of 4175-LuT exosome at a concentration of 1.5 × 10^6^/mL. The binding capacity of the IDAB probe is also verified with scanning electron microscopy before sensing experiment. When exosome contacts with IDAB modified surface, exosome can be observed under scanning electron microscopy, while the control group remains to be a clean surface. We observe in our sensing experiment that the pSPR phasogram from two ROIs in the vicinity again exhibit seemingly different results for the sensing of exosomes. However, the sensorgram data can be made highly consistent, once the mapping algorithm is applied. At the end of the exosome sensing, CD9 as a secondary antibody exhibited a strong signal with strong binding affinity, thereby verifying again the success of exosome detection on pSPR.

## Supplementary Materials

The following are available online at https://www.mdpi.com/2079-6374/11/3/95/s1.
