# Supplementary material for: Multi-Layer Reflectivity Calculation Based Meta-Modeling of the Phase Mapping Function for Highly Reproducible Surface Plasmon Resonance Biosensing
Source: Biosensors (Basel). 2021 Mar 23;11(3):95. doi: 10.3390/bios11030095 (PMC8004883; doi:10.3390/bios11030095)
Supplement: Supplementary file 1 [file biosensors-11-00095-s001.pdf]

biosensors

Supporting Information

Multi-layer Reflectivity Calculation Based Meta-Modeling of the Phase Mapping Function for Highly Reproducible Surface Plasmon Resonance Biosensing

Instruction of use of the algorithm code

The code of the algorithm can be executed with Spyder IDE with open source Anaconda or other Python IDE. The body of the code is comprised of two py file, i.e Meta-model.py and its library fLib.py. The Meta-model.py should be put in the same folder with fLib.py to properly executed the code, unless otherwise modified. Depending on the IDE environment, user may need to install packages needed for the execution. Those packages are noted in top of the fLib.py code.

Once executed, a User Interface will be shown, which consist of three major UI area (*Cf.* Fig.S1): Parameter and control panel, Data and Result display segment and an Event Log area. User will need to firstly import experimentally measured data in order to start optimization. It is also possible to manually input a set of Fresnel parameter and build corresponding function. This utility can help to allocate tuning range of the parameter before optimization process begin. Once the experimental data is loaded, user can press

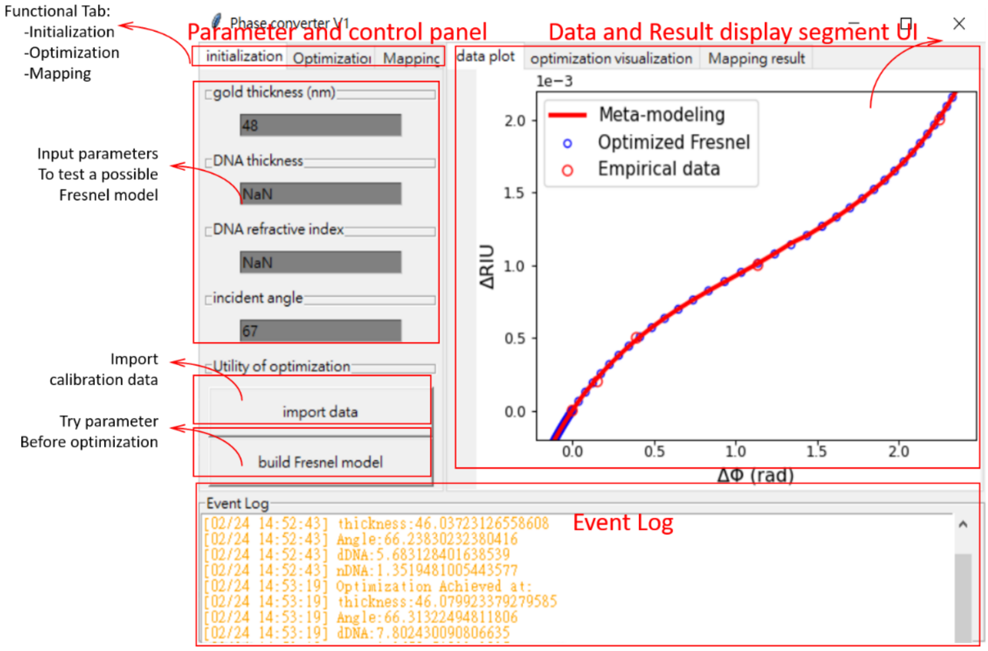

Figure S1. UI panel for the proposed algorithm.

"optimization" tab to commence optimization process as shown in Fig.S2. User will need to set the tuning range of the parameter, the number of evaluation rounds and then press the "optimization" button to search for the most-fit model. The fitting process can be monitored by the python console. Once the optimization is done, the optimized parameters will be displayed in the event log area. User can then proceed to press "optimization visualization "to check visualization chart.

When optimized meta-model is successfully built, user can proceed to import sensorgram data to convert phasogram. In order to do so, user will need to press "Mapping" tab (*Cf.* Fig.S3), and import the data correspondingly. The mapping result will be shown

in “Mapping result” tab. The converted sensorgram will automatically be saved under the working folder.

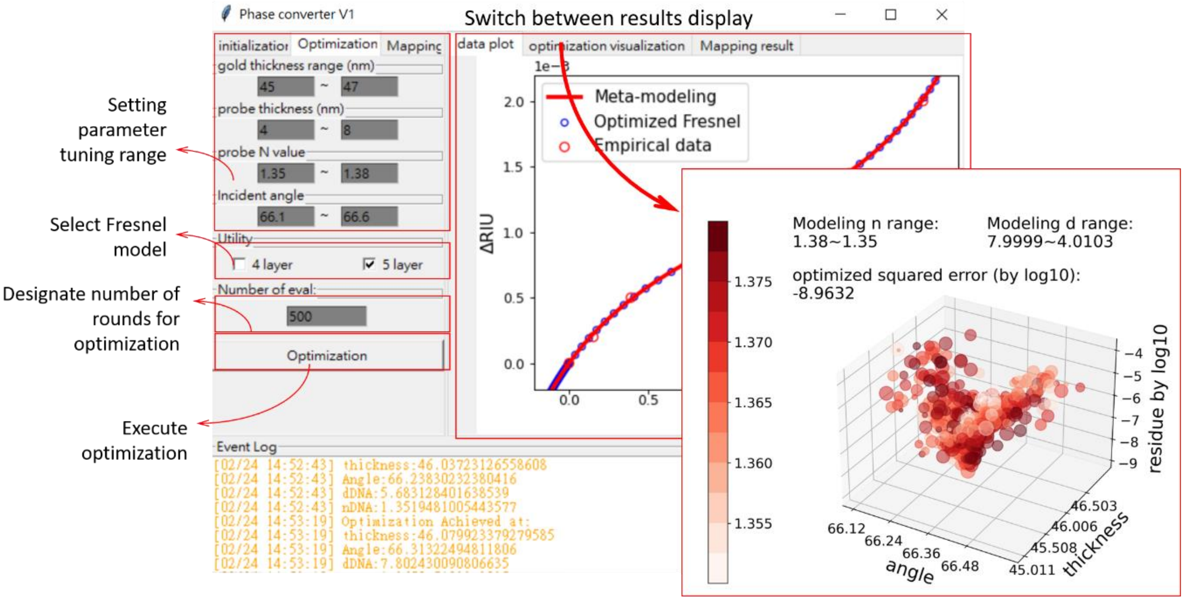

Figure S2. optimization UI pane.

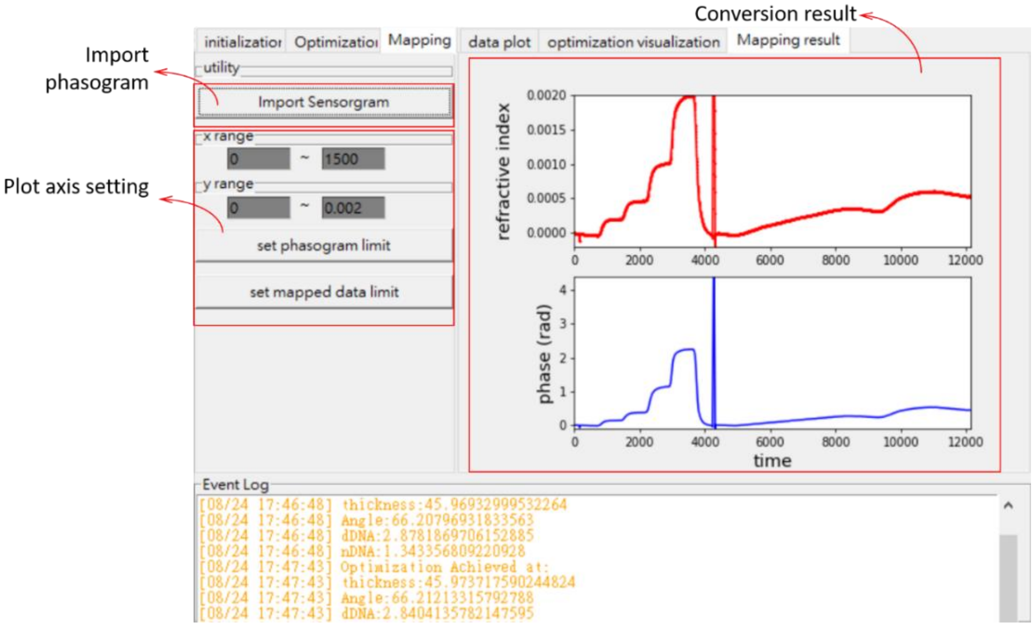

Figure S3. mapping function UI.
